# Interleukin‐6 and Oncostatin M Levels in Patients With Type 1 Diabetes and Its Relationship With Glycemic Indices

**DOI:** 10.1002/edm2.70179

**Published:** 2026-03-31

**Authors:** Mona Nourbakhsh, Farzaneh Rouhani, Maryam Razzaghy‐Azar, Mitra Nourbakhsh, Vahid Saeedi, Mahdokht Mehramiz, Davoud Amirkashani, Arash Mohazzab, Taraneh Rezaee

**Affiliations:** ^1^ Department of Pediatrics Endocrinology and Metabolism, School of Medicine, Ali Asghar Children's Hospital Iran University of Medical Sciences Tehran Iran; ^2^ Pediatric Growth and Development Research Center, Institute of Endocrinology and Metabolism Iran University of Medical Sciences Tehran Iran; ^3^ Department of Biochemistry, School of Medicine Iran University of Medical Sciences Tehran Iran; ^4^ School of Public Health Iran University of Medical Sciences Tehran Iran; ^5^ Department of Clinical Biochemistry, School of Medicine Iran University of Medical Sciences Tehran Iran

**Keywords:** diabetic ketoacidosis, glycemic indices, interleukin‐6, oncostatin M, paediatrics, type 1 diabetes mellitus

## Abstract

**Introduction:**

Type 1 diabetes mellitus (T1DM) is an autoimmune disorder characterised by progressive destruction of pancreatic β‐cells, in which inflammatory cytokines such as interleukin‐6 (IL‐6) and oncostatin M (OSM) play key roles. This study aimed to evaluate serum IL‐6 and OSM levels and their relationship with glycemic indices in children with T1DM.

**Methods:**

In this cross‐sectional study, 80 children (40 with T1DM and 40 controls) aged 3–18 years were included. Exclusion criteria were congenital or inflammatory disorders and use of anti‐inflammatory drugs, corticosteroids, NSAIDs, antibiotics, lipid‐lowering agents, hypoglycemic drugs, herbal supplements, multivitamins, or special diets. Serum IL‐6, OSM, fasting blood sugar (FBS), and HbA1c were measured. Demographic and clinical data—including age, sex, growth percentiles, BMI percentile, blood pressure, duration of diabetes, and history of diabetic ketoacidosis (DKA)—were recorded. Logistic regression was used to identify predictors of glycemic indices, and ROC curve analysis assessed the diagnostic performance of OSM. Statistical significance was set at *p* < 0.05.

**Results:**

Out of 80 subjects, the mean age was 10.62 (3.3) years in children with T1DM and 11.25 (3.6) years in controls, with a comparable gender distribution (male: 52.5% vs. 60%). Serum OSM levels were significantly higher in children with T1DM (*p* < 0.001) and strongly predicted elevated HbA1c (adjusted OR = 3.06; 95% CI: 1.84–5.08) and FBS (adjusted OR = 1.70; 95% CI: 1.31–2.20). ROC analysis demonstrated a robust ability to discriminate T1DM from controls (AUC = 0.94; 95% CI: 0.89–0.99), with a cut‐off of OSM > 10.0675, yielding a sensitivity and specificity of 87.5%. IL‐6 levels did not differ significantly between groups but were higher in children with a history of DKA.

**Conclusion:**

OSM may represent a sensitive and specific biomarker for distinguishing children with T1DM from healthy individuals, whereas IL‐6 appears more closely related to DKA‐associated inflammation.

## Graphical abstract

In children with type 1 diabetes, serum oncostatin M is significantly elevated and strongly predicts poor glycemic control, while IL‐6 is associated with diabetic ketoacidosis. OSM demonstrates high diagnostic accuracy, suggesting its potential as a sensitive biomarker for early detection and monitoring of paediatric T1DM.

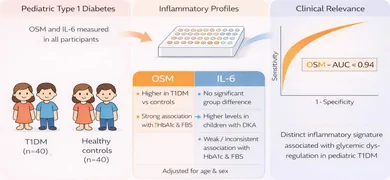

## 1 Introduction

Type 1 diabetes mellitus (T1DM) is a chronic condition characterised by a severe lack of insulin, requiring patients to use exogenous insulin to prevent ketosis and maintain life [1]. Over 1.2 million young people are living with this condition worldwide [2]. Globally, 108,300 new cases were reported in children under 15 years in 2021, increasing to 149,500 when including those under 20 years [3]. In the United States, the prevalence of T1DM among youths was 3.5 per 1000, with the highest rates observed in those aged 10 to 17 years (5.0 per 1000) [4]. Europe has the highest incidence rates, particularly in Finland, Sweden, and Kuwait, with 52.2, 44.1, and 41.7 per 100,000 person‐years, respectively [2]. In Asia, including South and East Asia and the MENA region, the incidence is lower but rising, with countries like China and Japan reporting 1.9 and 2.2 per 100,000 person‐years [2]. In Iran, the prevalence of T1DM among children and adolescents aged 0–19 years was estimated at 8.2 per 1000 in 2021, reflecting a growing burden in the region [5].

Some complications are related to T1DM, including ketoacidosis, hypoglycemia, retinal disorders, cardiovascular disorders, kidney involvement, and even death [6]. Diabetes imposes a substantial economic burden on the national healthcare systems, including hospitalisation for the treatment of the aforementioned complications, laboratory tests, and periodic visits to prevent damage caused by poor glycemic control and autoimmune complications associated with type 1 diabetes [7].

Oncostatin M (OSM) is a member of the GP130 cytokine family. It affects cell growth, neuronal development, and the inflammatory response by activating the JAK/STAT pathway [8]. OSM and its receptor are potential therapeutic targets in chronic inflammatory diseases, particularly those leading to endothelial dysfunction, such as rheumatoid arthritis, atherosclerosis, thrombosis, and abnormal angiogenesis. This cytokine helps maintain homeostasis in liver and adipose tissues; its absence can lead to obesity, hepatic steatosis, insulin resistance, and chronic inflammation [8].

Inflammatory mechanisms play a key role in the pathogenesis of type 1 diabetes, and certain cytokines, including members of the interleukin‐6 (IL‐6) family such as OSM, play a role in initiating inflammation and possibly contributing to disease complications [9]. The IL‐6 family has immune regulatory activity that directly affects glucose homeostasis. Cytokines of this family influence skeletal muscle cells, adipocytes, hepatocytes, and pancreatic beta cells, contributing to inflammation. The relationship between genetics and IL‐6 polymorphism in the development of type 1 and type 2 diabetes and insulin resistance has been determined [9]. Therefore, given the importance of inflammation and related biomarkers in paediatric type 1 diabetes, this study aims to evaluate IL‐6 and OSM levels and their association with glycemic indicators in children with T1DM. Our findings may help identify potential biomarkers for early diagnosis, risk assessment, and targeted therapy development.

## 2 Methods

### 2.1 Study Design

A cross‐sectional study was conducted between October 2023 and January 2024.

### 2.2 Setting

Using convenience sampling, participants were recruited from the endocrinology clinic of Ali Asghar Hospital in Tehran. This hospital provides a wide range of paediatric healthcare services within a comprehensive medical setting. This helps researchers to reach participants with a maximum variety of demographic and clinical characteristics.

### 2.3 Participants

Eighty participants were evaluated, including 40 children with type 1 diabetes and 40 non‐diabetic children. Children were eligible for inclusion if they (i) were of Iranian nationality, (ii) were between 3 and 18 years of age, and (iii) had no history of congenital diseases. Children were excluded if they had any inflammatory disease, or if they were taking medications that could interfere with OSM or IL‐6 measurements. Such medications included anti‐inflammatory drugs, corticosteroids, NSAIDs, antibiotics, hypoglycemic agents, lipid‐lowering medications (e.g., statins, fibrates, and nicotinic acid), or herbal medicines with uncertain biological effects. Children who were taking any medications or dietary supplements (including multivitamins), those with any underlying medical condition, children suspected of having growth hormone deficiency, or those following special diets such as milk‐based diets were also excluded from the study. Patients were asked to fulfil informed consent form. The study was conducted according to declaration of Helsinki (decree code: IR.IUMS.FMD.REC.1402.334).

### 2.4 Sampling Procedure

In endocrinology clinic, a list of names and contact number of mothers with a child aged 3–18 years were extracted. They were called and invited to participate in the study by explaining the study aims, and assured of the confidentiality of their data. Blood samples were collected from the children following standardised procedures and laboratory protocols. In addition, study questionnaires were administered and completed with the assistance of trained interviewers to ensure accuracy and consistency.

### 2.5 Study Size

The sample size was estimated using PASS 2022 software based on the study by Darvish et al. [10]. Considering a 40% difference between the healthy and case groups, a mean serum interleukin‐6 level of 5.45 with a standard deviation of 2.5, an effect size of 0.2, a type I error of 0.05, and a type II error of 0.2, the required sample size was calculated to be 40 participants in each group.

### 2.6 Measurement

Age, gender, height percentile, weight percentile, BMI percentile, duration of diabetes, presence or absence of DKA, total daily insulin dose, and systolic and diastolic blood pressure of cases were obtained via history taking and physical examination.

A 5 mL venous blood sample was collected from each participant. The samples were then transported to the Colife Laboratory for biochemical analysis, including measurement of serum IL‐6, HbA1c, FBS, and OSM. Serum concentrations of IL‐6 and OSM were quantified using commercially available enzyme‐linked immunosorbent assay (ELISA) kits (R&D System, Germany), according to the manufacturer's instructions.

### 2.7 Statistical Analysis

Data were analysed using SPSS version 26 (IBM crop, New York, USA) and Stata version 14. Continuous data were summarised using means and standard deviations (SDs), and categorical variables were presented using frequencies and percentages. The Kolmogorov–Smirnov test was applied to investigate the normality of quantitative variables to use parametric tests. Univariable and multivariable logistic regression were conducted. In the logistic regression method, the response variable should be the two‐state qualitative type. To identify the predictive role of structural selected determinants for different domains of HbA1c and FBS, univariable logistic regression was conducted. The significance value for multivariable logistic regression was set at *p* < 0.05.

## 3 Results

A total of 80 individuals were included in the present study. Demographic and clinical characteristics of participants are provided in Table 1. The gender distribution was also similar, with a slightly higher proportion of boys in both groups. Most participants in both groups fell within the normal BMI‐for‐age percentile range, although obesity was more frequent among patients with type 1 diabetes (12.5%). Additionally, 45% of the diabetic group had a history of diabetic ketoacidosis. The mean values of FBS and HbA1c were 222.03 (SD = 96.37) mM/L and 9.59 (SD = 2.12) in individuals with type 1 diabetes, and 95.38 (SD = 18.02) mM/L and 5.44 (SD = 0.30) in individuals without type 1 diabetes, respectively.

**TABLE 1:** Demographic and clinical characteristics of individuals with and without diabetes.

Baseline characteristics	Individuals with diabetes (*N* = 40)	Individuals without diabetes (*N* = 40)
Age (years), mean (SD)	10.62 (3.3)	11.25 (3.6)
Gender, *N* (%)		
Boy	21 (52.5)	24 (60.0)
Girl	19 (47.5)	16 (40.0)
BMI‐for‐age percentiles, *N* (%) (kg/m^2^)		
Underweight (< 5th percentile)	2 (5.0)	3 (7.5)
Normal (5th–85th percentile)	21 (52.5)	29 (72.5)
Overweight (85th–95th percentile)	4 (10.0)	4 (10.0)
Obese (≥ 95th percentile)	5 (12.5)	49.9 (9.9)
Thyroid disease, *N* (%)		
Yes	3 (7.5)	2 (5.0)
No	37 (92.5)	38 (95.0)
Celiac disease, *N* (%)		
Yes	1 (2.5)	0
No	39 (97.5)	40 (100.0)
DKA, *N* (%)		
Yes	18 (45.0)	—
No	22 (55.0)	—
Systolic blood pressure (mean/SD)	56 (14.5)	51 (6.3)
Systolic blood pressure (mean/SD)	58 (16.2)	50 (0)
FBS/mg/dL (mean/SD)	222.03 (96.37)	95.38 (18.02)
HbA1c (mean/SD)	9.59 (2.12)	5.44 (0.30)
IL6 (pg/mL) (mean/SD)	13.09 (44.82)	2.37 (2.27)
OSM (pg/mL) (mean/SD)	12.2 (2.41)	7.49 (2.69)

Abbreviations: BMI, body mass index; DKA, diabetic ketoacidosis; FBS, fasting blood sugar; OSM, oncostatin M.

The predictive role of demographic and clinical characteristics for HbA1c and FBS via uni‐variable and multivariable logistic regression analysis are provided in Table 2. Elevated HbA1c levels were almost three times higher among children with increased OSM levels (aOR: 3.06, 95% CI: 1.84; 5.08), and 1% higher with each unit increase in log IL‐6 concentration (aOR: 1.01, 95% CI: 1.00; 1.04). FBS levels were 2% higher among children with increased log IL‐6 concentration (aOR: 1.02, 95% CI: 1.0; 1.06), and 70% higher among children with increased oncostatin M levels (aOR: 1.70, 95% CI: 1.31; 2.20).

**TABLE 2:** Univariate and multivariate analysis of factors associated with HbA1c and FBS in type 1 diabetes.

Predictors	HbA1c*		FBS**	
	Crude OR (95% CI)	Adjusted OR (95% CI)	Crude OR (95% CI)	Adjusted OR (95% CI)
Age	1.00 (0.98, 1.00)	1.00 (0.98, 1.01)	0.99 (0.98, 1.00)	0.99 (0.98, 1.00)
Gender (ref: boy)	1.56 (0.64, 3.81)	1.60 (0.65, 3.95)	1 (0.41, 2.46)	1.06 (0.42, 2.68)
BMI‐for‐age percentiles				
Underweight	Reference group		Reference group	
Normal	0.77 (0.11, 5.07)	0.58 (0.08, 4.10)	0.84 (0.13, 5.53)	0.71 (0.10, 5.10)
Overweight	2.5 (0.32, 19.53)	1.32 (0.15, 11.34)	1.5 (0.19, 11.54)	1.32 (0.15, 11.34)
Obese	1.88 (0.20, 17.27)	0.90 (0.09, 9.04)	1.2 (0.13, 11.05)	0.89 (0.9, 9.04)
Log IL6 (pg/mL)	1.31 (0.78, 2.18)	**1.25 (1.02, 2.11)**	**1.46 (1.01, 2.51)**	**1.38 (1.00, 2.46)**
OSM (pg/mL)	**2.96 (1.81, 4.83)**	**3.06 (1.84, 5.08)**	**1.60 (1.30, 2.02)**	**1.70 (1.31, 2.20)**

*Note:* Adjusted by age and gender. Bold values indicate statistically significant associations (*P* < 0.05).

Abbreviations: BMI, body mass index; IL6, interleukin 6; OSM, oncostatin M.

* Ref: ≤ 7.5%.

** Ref: ≤ 130 mg/dL.

**FIGURE 1:**
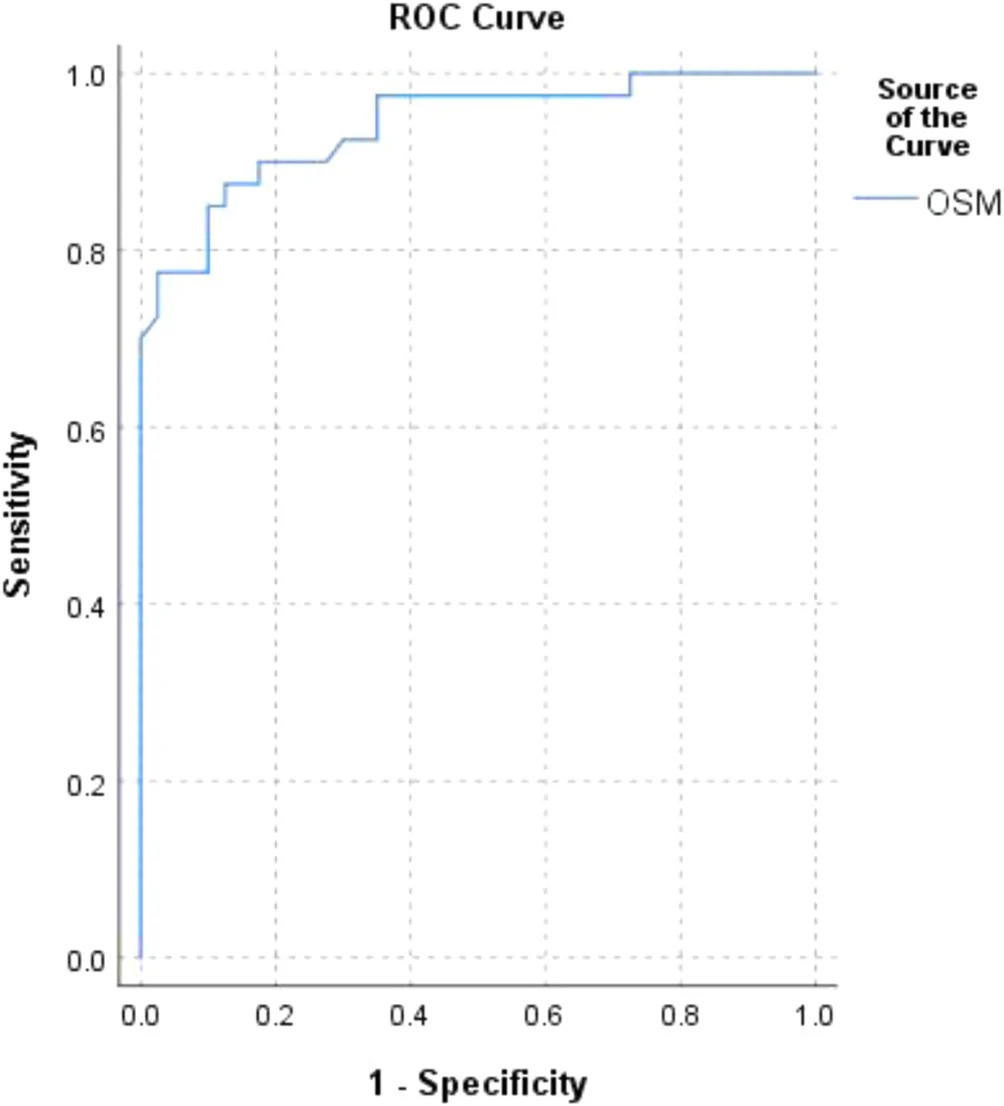
ROC curve analysis for diagnostic accuracy of OSM in type 1 diabetes diagnosis.

The analysis of the ROC curve showed that the serum level of OSM is a good indicator for diagnosing type 1 diabetes in healthy people (the area under the ROC curve: 0.94; 95% CI [0.89, 0.99]). Also, ROC analysis showed that the best cut‐off with the best sensitivity and specificity for diagnosing type 1 diabetes in children was equal to OSM > 10.0675 with a sensitivity of 87.5% and a specificity of 87.5% (Figure 1).

## 4 Discussion

We investigated the role of inflammatory biomarkers, oncostatin M (OSM) and IL‐6, in children with type 1 diabetes and assessed their association with glycemic indicators. Our findings showed that, as expected, children with type 1 diabetes exhibited higher glycemic indices compared with healthy individuals, which aligns with the known pathophysiology of impaired insulin production in type 1 diabetes. We identified two key findings.

First, higher circulating levels of both OSM and IL‐6 were meaningfully associated with elevated HbA1c and fasting blood glucose, supporting the hypothesis that inflammatory pathways may contribute to metabolic dysregulation in children with type 1 diabetes. Both cytokines play important roles in immune‐mediated pathways: IL‐6 is known to influence β‐cell function, insulin sensitivity, and hepatic glucose output, while OSM—another member of the IL‐6 cytokine family—has been linked to inflammatory signalling, altered insulin action, and metabolic stress in adipose and muscle tissues [11, 12]. Their concurrent elevation alongside poorer glycemic markers may therefore reflect heightened systemic inflammation, increased β‐cell burden, or immune activation related to hyperglycemia.

These findings are partly aligned with previous research. Several studies have reported that IL‐6 correlates positively with HbA1c and fasting glucose in individuals with type 1 diabetes, suggesting that IL‐6 may rise in response to chronic hyperglycemia or ongoing autoimmune activity [13, 14]. However, other studies have found no such association, possibly due to differences in age, disease duration, insulin regimens, or inflammatory status of the comparison groups. Evidence regarding OSM in type 1 diabetes is extremely limited, yet data from metabolic and obesity‐related studies indicate that OSM can impair insulin signalling and promote metabolic dysregulation, supporting the plausibility of our findings [15, 16].

Support for our findings comes from previous studies examining the role of OSM in insulin resistance. For instance, Akarsu et al. [8] investigated 50 individuals with insulin resistance (IR) and 34 healthy controls and reported that serum OSM levels were significantly correlated with IR indices such as HOMA‐IR and QUICKI. Similarly, Sanchez‐Infantes et al. [15] demonstrated that OSM produced by adipose tissue macrophages correlates with insulin levels and impaired glucose disposal in humans. At the cellular level, Bailey et al. [16] showed that OSM induces lipolysis and reduces insulin responsiveness in adipocytes, while Moreno et al. [17] reported that neutralisation of OSM in obese mice improved glucose metabolism, and in humans, OSM levels correlated with systemic inflammation and insulin resistance. Although most of these studies were conducted in adults or in metabolic syndrome/obesity models rather than paediatric T1D, they collectively support the plausibility of our results, indicating that OSM may contribute to inflammatory pathways underlying metabolic dysregulation.

Second, OSM demonstrated high overall diagnostic performance, with sensitivity and specificity values that were both high and identical, indicating that this cytokine may serve as a reliable biomarker for distinguishing children with type 1 diabetes from healthy individuals. Currently, clinical studies directly evaluating the diagnostic performance of OSM in type 1 diabetes are very limited. Most existing evidence comes from research on obesity and type 2 diabetes, where elevated OSM levels have been associated with insulin resistance, impaired glucose metabolism, and systemic inflammation. While these studies suggest a biological plausibility for OSM's role in metabolic dysregulation, our findings are among the first to demonstrate its potential as a sensitive and specific biomarker for distinguishing children with type 1 diabetes from healthy peers. These results highlight the promise of OSM for early detection, though further studies with larger cohorts are needed to confirm its clinical utility and establish standardised diagnostic thresholds.

In addition to the findings of the present study, substantial evidence from previous investigations in paediatric populations with type 1 diabetes supports the role of systemic inflammation in impaired glycemic control. Several studies have reported significantly higher serum levels of interleukin‐6 (IL‐6) in children with type 1 diabetes compared with healthy controls, with this increase often being associated with higher HbA1c levels and poorer glycemic control. For example, Fawaz et al. demonstrated that IL‐6 and C‐reactive protein levels were significantly elevated in children with type 1 diabetes, particularly among those with poor glycemic control [18]. Similarly, Hammed et al. reported significantly increased IL‐6 concentrations in paediatric patients with type 1 diabetes, showing positive and significant correlations with both HbA1c and fasting blood glucose [19]. These findings support the hypothesis that chronic inflammation may be linked to the severity of metabolic dysregulation in children with type 1 diabetes.

However, not all studies have reported consistent results. Some investigations, particularly in children with longer disease duration or more stable metabolic control, have found no significant differences in IL‐6 levels between patients and healthy individuals. Wedrychowicz et al. showed that IL‐6 levels are elevated at the time of initial diagnosis of type 1 diabetes but tend to return to levels comparable to those of healthy controls during the chronic phase of the disease, with no significant association with HbA1c [20]. Likewise, Fahad et al. reported increased IL‐6 levels in children with type 1 diabetes but did not observe a significant correlation between IL‐6 and glycemic indices [21]. Such discrepancies may be attributed to differences in patient age, disease duration, glycemic control status, or the presence of acute inflammatory conditions such as diabetic ketoacidosis.

The findings of the present study are partially consistent with these observations. Although IL‐6 levels did not differ significantly between children with type 1 diabetes and healthy controls, higher IL‐6 concentrations were observed in patients with a history of diabetic ketoacidosis. This finding suggests that IL‐6 may reflect acute inflammatory and severe metabolic stress states rather than serving as a stable biomarker of chronic glycemic control in paediatric type 1 diabetes.

In contrast, data regarding the role of oncostatin M (OSM) in paediatric type 1 diabetes are extremely limited, and the present study is among the first to investigate this cytokine in this population. Our results demonstrated that serum OSM levels were significantly higher in children with type 1 diabetes compared with healthy individuals and showed a strong and independent association with both HbA1c and fasting blood glucose levels. Although direct clinical studies in children with type 1 diabetes are lacking, evidence from metabolic studies in other populations supports our findings. Akarsu et al. reported elevated OSM levels in individuals with insulin resistance, with significant correlations between OSM and insulin resistance indices [8]. Furthermore, studies in obesity and metabolic disorders have shown that OSM may contribute to glucose dysregulation through impaired insulin signalling, increased lipolysis, and enhanced tissue inflammation [15, 16, 17]. The differential patterns observed for IL‐6 and OSM in the present study may reflect distinct biological roles of these cytokines in paediatric type 1 diabetes. While IL‐6 appears to be more closely related to transient or acute inflammatory states, OSM may represent a more persistent inflammatory pathway associated with chronic glucose dysregulation. This distinction may explain the superior diagnostic performance of OSM compared with IL‐6 in discriminating children with type 1 diabetes from healthy controls.

Overall, comparison of the present findings with previous evidence suggests that although inflammation plays a critical role in the pathophysiology of paediatric type 1 diabetes, not all inflammatory biomarkers have equal clinical value. Our results indicate that oncostatin M may serve as a promising biomarker for the identification and monitoring of type 1 diabetes in children, whereas IL‐6 may primarily reflect acute inflammatory burden or the occurrence of complications such as diabetic ketoacidosis. Longitudinal studies with larger sample sizes are warranted to confirm these findings and to clarify the clinical utility of these cytokines.

## 5 Limitations

Despite the important findings, this study has several limitations. First, the cross‐sectional design precludes establishing causality between elevated OSM or IL‐6 levels and glycemic dysregulation. Second, the relatively small sample size may limit the generalizability of the results to broader paediatric populations. Third, we measured OSM and IL‐6 at a single time point, which may not fully reflect fluctuations in cytokine levels related to disease activity or glycemic control over time. Additionally, potential confounding factors such as diet, physical activity, and concomitant subclinical infections were not fully controlled. Finally, as clinical studies examining OSM in type 1 diabetes are scarce, there is limited external data for comparison, and further longitudinal research is needed to validate our findings and clarify the utility of OSM as a diagnostic biomarker.

## 6 Conclusion

Our study highlights the potential role of inflammatory biomarkers, particularly oncostatin M (OSM) and IL‐6, in the pathophysiology of paediatric type 1 diabetes. We demonstrated that higher circulating levels of these cytokines were associated with elevated glycemic indices, suggesting a link between systemic inflammation and metabolic dysregulation. Importantly, OSM showed strong diagnostic potential, with high sensitivity and specificity for distinguishing children with type 1 diabetes from healthy peers, indicating its promise as a reliable biomarker. These findings underscore the relevance of inflammatory pathways in type 1 diabetes and support further investigation of OSM and IL‐6 as tools for early diagnosis, risk stratification, and potential therapeutic targeting in paediatric populations.

## Author Contributions

**Mona Nourbakhsh:** Conceptualization, Project Administration. **Farzaneh Rouhani:** Conceptualization, Revise and Editing. **Maryam Razzaghy‐Azar:** Conceptualization, Supervision, reviewing and editing manuscript. **Mitra Nourbakhsh:** Biochemical measurements, Methodology. **Vahid Saeedi:** data curation, Clinical assessment. **Mahdokht Mehramiz:** Data curation, Data interpretation, Original Draft Preparation, Revise and Editing, Correspondence. **Davoud Amirkashani:** Data interpretation, Methodology, Revise and Editing. **Arash Mohazzab:** Statistical analysis, Formal analysis, Data interpretation. **Taraneh Rezaee:** Biochemical measurements, Methodology.

## Funding

The authors have nothing to report.

## Ethics Statement

This study was approved by the Ethics Committee of Iran University of Medical Sciences (IR.IUMS.FMD.REC.1402.334). All procedures were performed in accordance with the ethical standards of the institutional review board and the Declaration of Helsinki.

## Consent

Written informed consent was obtained from all participants or their legal guardians before inclusion in the study.

## Conflicts of Interest

The authors declare no conflicts of interest.

## Data Availability

The datasets generated and analysed during the current study are available from the corresponding author upon reasonable request. No publicly accessible database was used for this research.

## References

[1] M. A. Sperling , Sperling Pediatric Endocrinology, 5th ed. (Elsevier Health Sciences, 2020).

[2] I. Hormazábal‐Aguayo , Y. Ezzatvar , N. Huerta‐Uribe , R. Ramírez‐Vélez , M. Izquierdo , and A. García‐Hermoso , “Incidence of Type 1 Diabetes Mellitus in Children and Adolescents Under 20 Years of Age Across 55 Countries From 2000 to 2022: A Systematic Review With Meta‐Analysis,” Diabetes, Metabolic Syndrome and Obesity: Targets and Therapy 40, no. 3 (2024): e3749. 10.1002/dmrr.3749 38037806

[3] G. D. Ogle , S. James , D. Dabelea , et al., “Global Estimates of Incidence of Type 1 Diabetes in Children and Adolescents: Results From the International Diabetes Federation Atlas, 10th Edition,” Diabetes Research and Clinical Practice 183 (2022): 109083. 34883188 10.1016/j.diabres.2021.109083

[4] M. Fang , D. Wang , and E. Selvin , “Prevalence of Type 1 Diabetes Among US Children and Adults by Age, Sex, Race, and Ethnicity,” JAMA 331, no. 16 (2024): 1411–1413. 38573653 10.1001/jama.2024.2103 PMC11040401

[5] M. Ghojazadeh , M. Mobasseri , F. P. Azar , and A. Lotfi , “Prevalence and Incidence of Type 1 Diabetes in the World,” (2024).

[6] S. Foti Randazzese , M. La Rocca , B. Bombaci , et al., “Severe Diabetic Ketoacidosis in Children With Type 1 Diabetes: Ongoing Challenges in Care,” Children 12, no. 1 (2025): 110. 39857941 10.3390/children12010110 PMC11763767

[7] J. C. Simeone , S. Shah , M. L. Ganz , et al., “Healthcare Resource Utilization and Cost Among Patients With Type 1 Diabetes in the United States,” Journal of Managed Care & Specialty Pharmacy 26, no. 11 (2020): 1399–1410. 33119443 10.18553/jmcp.2020.26.11.1399 PMC10390920

[8] M. Akarsu , M. Hurşitoğlu , Z. Toprak , et al., “Relationships Among Oncostatin M, Insulin Resistance, and Chronic Inflammation: A Pilot Study,” Archives of Endocrinology and Metabolism 64, no. 1 (2020): 38–44. 31576964 10.20945/2359-3997000000176 PMC10522293

[9] J. Liu , T. Wang , F. Yang , and C. Pubu , “Advancements in the Understanding of Mechanisms of the IL‐6 Family in Relation to Metabolic‐Associated Fatty Liver Disease,” Frontiers in Endocrinology 16 (2025): 1642436. 40969371 10.3389/fendo.2025.1642436 PMC12440729

[10] A. H. Darwish , T. M. Elgohary , and N. A. Nosair , “Serum Interleukin‐6 Level in Children With Attention‐Deficit Hyperactivity Disorder (ADHD),” Journal of Child Neurology 34, no. 2 (2019): 61–67. 30430896 10.1177/0883073818809831

[11] M. Akbari and V. Hassan‐Zadeh , “IL‐6 Signalling Pathways and the Development of Type 2 Diabetes,” Inflammopharmacology 26, no. 3 (2018): 685–698. 29508109 10.1007/s10787-018-0458-0

[12] M. D. Giraldez , D. Carneros , C. Garbers , S. Rose‐John , and M. Bustos , “New Insights Into IL‐6 Family Cytokines in Metabolism, Hepatology and Gastroenterology,” Nature Reviews Gastroenterology & Hepatology 18, no. 11 (2021): 787–803. 34211157 10.1038/s41575-021-00473-x

[13] A. S. Syed Khaja , N. K. Binsaleh , M. M. A. Beg , et al., “Clinical Importance of Cytokine (IL‐6, IL‐8, and IL‐10) and Vitamin D Levels Among Patients With Type‐1 Diabetes,” Scientific Reports 14, no. 1 (2024): 24225. 39414864 10.1038/s41598-024-73737-6 PMC11484771

[14] T. Koufakis , D. Kouroupis , A. Kourti , et al., “Interleukin‐6‐Related Inflammatory Burden in Type 1 Diabetes: Evidence for Elevation With Suboptimal Glycemic Control,” Journal of Clinical Medicine 14, no. 18 (2025): 6511. 41010714 10.3390/jcm14186511 PMC12471011

[15] D. Sanchez‐Infantes , U. A. White , C. M. Elks , et al., “Oncostatin m Is Produced in Adipose Tissue and Is Regulated in Conditions of Obesity and Type 2 Diabetes,” Journal of Clinical Endocrinology and Metabolism 99, no. 2 (2014): E217–E225. 24297795 10.1210/jc.2013-3555 PMC3913819

[16] J. L. Bailey , H. Hang , A. Boudreau , and C. M. Elks , “Oncostatin M Induces Lipolysis and Suppresses Insulin Response in 3T3‐L1 Adipocytes,” International Journal of Molecular Sciences 23, no. 9 (2022): 4689. 35563078 10.3390/ijms23094689 PMC9104719

[17] I. Piquer‐Garcia , L. Campderros , S. D. Taxerås , et al., “A Role for Oncostatin M in the Impairment of Glucose Homeostasis in Obesity,” Journal of Clinical Endocrinology and Metabolism 105, no. 3 (2020): e337–e348. 31606738 10.1210/clinem/dgz090 PMC7112982

[18] L. Fawaz , A. E. Elwan , Y. H. Kamel , T. M. Farid , A. Kamel , and W. A. Mohamed , “Value of C‐Reactive Protein and IL‐6 Measurements in Type 1 Diabetes Mellitus,” Archives of Medical Science 5, no. 3 (2009): 383–390.

[19] I. K. Hammed , N. F. Rashid , and B. A. Abed , “Serum Interleukin‐6 Level in Children With Type 1 Diabetes Mellitus,” Journal of the Faculty of Medicine Baghdad 54, no. 3 (2012): 228–230.

[20] A. Wedrychowicz , H. Dziatkowiak , K. Sztefko , and A. Wedrychowicz , “Interleukin‐6 (IL‐6) and IGF‐IGFBP System in Children and Adolescents With Type 1 Diabetes Mellitus,” Experimental and Clinical Endocrinology & Diabetes 112, no. 8 (2004): 435–439. 15372363 10.1055/s-2004-821189

[21] H. M. Fahad , A. N. Jassim , and S. M. Nada , “Investigation of Inflammatory Markers (IL‐6 and Hs‐CRP) in Type1 Diabetes Patients,” International Journal of Advanced Research 2, no. 3 (2014): 572–579.

